# Editorial: The 17th Annual *Nucleic Acids Research* Web Server Issue 2019

**DOI:** 10.1093/nar/gkz521

**Published:** 2019-06-28

The 2019 Web Server Issue of Nucleic Acids Research is the 17th in a series of annual issues dedicated to web-based software resources for analysis and visualization of molecular biology data. It is freely available online under NAR’s open access policy. This year, 331 proposals were submitted and 122, or 37%, were approved for manuscript submission. Of those approved, 94, or 77%, were ultimately accepted for publication. Table 1 lists the 2019 Web Servers, their URLs and a brief description of each.

**Table 1.** Descriptions of Web Servers in the *NAR* 2019 Web Server Issue

Web Server name	URL	Brief description
**2StrucCompare**	http://2struccompare.cryst.bbk.ac.uk/index.php	Visualization of differences between protein structures
**Aggrescan3D 2.0**	http://biocomp.chem.uw.edu.pl/A3D2/	Prediction of protein solubility
**AlloDriver**	http://mdl.shsmu.edu.cn/ALD	Identification of targets for cancer driver mutations
**antiSMASH 5.0**	https://antismash.secondarymetabolites.org	Secondary metabolite biosynthetic gene clusters
**AppA**	http://mspc.bii.a-star.edu.sg/minhn/appa.html	Antibody–antigen structures and models
**AutoMLST**	https://automlst.ziemertlab.com	Bacterial multi-locus sequence analysis and species trees
**BEERE**	http://discovery.informatics.uab.edu/beere/	Search for relationships between genes, proteins and biomedical terms
**BioUML**	http://ict.biouml.org	Integrated computational platform for systems biology
**CaverWeb**	https://loschmidt.chemi.muni.cz/caverweb	Analysis of protein tunnels and channels
**ChEA3**	https://amp.pharm.mssm.edu/ChEA3	Transcription factor enrichment analysis
**CHOPCHOP v3**	http://valenvm.cbu.uib.no/	CRISPR-Cas web toolbox
**Cistrome-GO**	http://go.cistrome.org/	ChIP-seq transcription factor gene target functional enrichment
**CNIT**	http://cnit.noncode.org/CNIT	Protein-coding and long non-coding RNA transcript classification
**CPGAVAS2**	http://www.herbalgenomics.org/cpgavas2	Plastome genomes annotation and analysis
**DaReUS-Loop**	http://bioserv.rpbs.univ-paris-diderot.fr/services/DaReUS-Loop/	Protein structure loop modeling
**DNAvisualization.org**	https://DNAvisualization.org	DNA 2D graphical visualizations
**Doc2Hpo**	https://impact2.dbmi.columbia.edu/doc2hpo/	Find HPO terms (Human Phenotype Ontology) by text mining
**DOGMA**	https://domainworld-services.uni-muenster.de/dogma/	Proteome and transcriptome quality assessment
**DrReposER**	http://mfrlab.org/drugreposer/	Identification of targets for drug repurposing
**DrugComb**	https://drugcomb.fimm.fi	Drug combination analysis for cancer cell lines
**EMBL-EBI APIs**	https://bit.ly/EMBL-EBI-APIs	Web services for bioinformatics sequence analysis applications and text search
**EpiAlignment**	https://epialign.ucsd.edu/	Genomic region alignment by sequence and epigenomic information
**EPIC-TABSAT**	https://tabsat.ait.ac.at/	Analysis of targeted bisulfite sequencing and array-based methylation studies
**Evolview v3**	https://www.evolgenius.info/evolview/	Phylogenetic tree visualization
**FidoSNP**	http://fidosnp.bca.unipd.it/	DNA variant effect prediction in dogs
**g:Profiler**	https://biit.cs.ut.ee/gprofiler	Gene set functional enrichment analysis
**GalaxyRefine2**	http://galaxy.seoklab.org/refine2	Protein structure refinement
**Gene Sculpt Suite**	http://www.genesculpt.org	Genome editing and engineering
**Geneshot**	https://amp.pharm.mssm.edu/geneshot	Ranking genes from text queries
**GEPIA2**	http://gepia2.cancer-pku.cn/	RNAseq expression analysis
**HawkDock**	http://cadd.zju.edu.cn/hawkdock/	Prediction of protein–protein binding structures
**HNADOCK**	http://huanglab.phys.hust.edu.cn/hnadock/	Modeling RNA/DNA complex structures
**IAMBEE**	http://bioinformatics.intec.ugent.be/iambee/	Genotype–phenotype mapping of clonal populations
**IEDB-AR**	http://tools.iedb.org/	Prediction and analysis of B- and T-cell epitopes
**iMKT**	https://imkt.uab.cat/	McDonald and Kreitman statistical test for evolution positive selection
**ImmuneRegulation**	https://immuneregulation.mssm.edu/	Human immune regulatory elements
**INGA2**	https://inga.bio.unipd.it/	Protein function prediction for the dark proteome
**IntFOLD**	http://www.reading.ac.uk/bioinf/IntFOLD/	Protein structure and function prediction
**iTOL v4**	https://itol.embl.de	Visualization and annotation of phylogenetic trees
**LitSense**	https://www.ncbi.nlm.nih.gov/research/litsense	Text mining sentence retrieval PubMed
**LnCompare**	http://www.rnanut.net/lncompare/	Feature analysis for human long noncoding RNAs
**LOMETS2**	https://zhanglab.ccmb.med.umich.edu/LOMETS/	Template-based protein structure prediction
**M1CR0B1AL1Z3R**	https://microbializer.tau.ac.il/	Microbial genomics analysis
**MAFFT-DASH**	https://mafft.cbrc.jp/alignment/server/	Multiple alignment protein sequence and structure
**mCSM-PPI2**	http://biosig.unimelb.edu.au/mcsm_ppi2/	Prediction of the effects of mutations on protein–protein interactions
**MERMAID**	http://molsim.sci.univr.it/mermaid/	Molecular dynamics coarse grained membrane proteins
**MEXPRESS**	https://mexpress.be/	Gene expression, DNA methylation and clinical TCGA data
**MFEprimer-3.0**	https://www.mfeprimer.com/	Quality control for PCR primers
**MISIM v2.0**	http://www.lirmed.com/misim/	MicroRNA functional similarity
**modEnrichr**	http://amp.pharm.mssm.edu/modEnrichr/	Gene set enrichment for model organisms
**MRPrimerW2**	http://mrprimerw2.com/	Primer design for qPCR
**MS^2^PIP**	https://iomics.ugent.be/ms2pip/	Prediction of tandem mass spectrometry signal peak intensities from peptide sequences
**MTR-Viewer**	http://biosig.unimelb.edu.au/mtr-viewer/	Genetic missense tolerance
**MutationDistiller**	https://www.mutationdistiller.org/	Phenotype-based search for disease mutations
**MyDGR**	http://omics.informatics.indiana.edu/myDGR	Diversity-generating retroelements
**NAPS**	http://bioinf.iiit.ac.in/NAPS/	Network analysis of protein structures
**NetGO**	http://issubmission.sjtu.edu.cn/netgo/	Protein function prediction
**NetworkAnalyst 3.0**	https://www.networkanalyst.ca	Gene expression profiling
**NGPhylogeny.fr**	https://ngphylogeny.fr	Phylogenetic analysis
**OGDRAW**	https://chlorobox.mpimp-golm.mpg.de/OGDraw.html	Visualization of organellar genomes (plastids and mitochondria)
**OrthoVenn2**	https://orthovenn2.bioinfotoolkits.net/	Analysis of orthologous gene clusters
**ORVAL**	https://orval.ibsquare.be	Prediction of disease-causing oligogenic variant combinations
**PatchSearch**	http://bioserv.rpbs.univ-paris-diderot.fr/services/PatchSearch	Off-target protein region search
**Pergola-web**	https://pergola.crg.eu/	Conversion of longitudinal behavioral data into genomic data formats
**Phylogeny.IO**	https://phylogeny.io	Phylogenetic tree visualization
**PIZSA**	http://cospi.iiserpune.ac.in/pizsa	Protein–protein interactions
**PrankWeb**	http://prankweb.cz/	Ligand-binding site prediction
**PRECOG**	http://precog.russelllab.org	G-protein coupled receptors protein coupling probabilities
**Prophage Hunter**	https://pro-hunter.bgi.com/	Search for active prophages in bacterial DNA
**ProSNEx**	http://prosnex-tool.com	Protein structure network residue interaction
**PSICA**	http://qas.wangwb.com/∼wwr34/mufoldqa/index.html	Protein structure model quality analysis
**PSIPRED**	http://bioinf.cs.ucl.ac.uk/psipred	Protein structure prediction and analysis
**PubTator central**	https://www.ncbi.nlm.nih.gov/research/pubtator/	Concept annotation in PubMed abstracts and PMC full-text articles
**QBiC-Pred**	http://qbic.genome.duke.edu	Effect of non-coding mutations on transcription factor binding
**RegulationSpotter**	https://www.regulationspotter.org/	Disease-causing potential of extragenic DNA variants
**ResponseNet v.3**	http://netbio.bgu.ac.il/respnet	Signaling and regulatory pathway analysis
**RNA workbench 2.0**	https://rna.usegalaxy.eu	RNA analysis tools
**RNAmod**	https://bioinformatics.sc.cn/RNAmod/	Annotation of mRNA modifications
**SEanalysis**	http://licpathway.net/SEanalysis	Super-enhancers gene regulatory networks
**SEPPA 3.0**	http://bidd2.nus.edu.sg/SEPPA3/	Epitope prediction of glycoprotein antigens
**SeqTailor**	http://shiva.rockefeller.edu/SeqTailor/	Sequence extraction for genomic variants and genomic intervals
**SimpleClinVar**	http://simple-clinvar.broadinstitute.org/	Visual exploration of ClinVar mutations
**SPADE**	https://spade.uni-graz.at/	Prediction of allergen IgE epitopes
**sRNAbench and sRNAtoolbox 2019**	https://arn.ugr.es/srnatoolbox/	Small RNA analysis tools
**SwissTargetPrediction**	http://www.swisstargetprediction.ch/	Prediction of protein targets of small molecules
**tRNAviz**	http://trna.ucsc.edu/tRNAviz/	Comparative analysis of tRNA sequence information
**VOLPES**	http://volpes.univie.ac.at/	Visualization of physicochemical properties of biological sequences
**VoroMQA**	http://bioinformatics.ibt.lt/wtsam/voromqa	Estimation of protein structure quality
**WashU Epigenome Browser update 2019**	https://epigenomegateway.wustl.edu/browser/	Investigation of epigenomic datasets
**Web 3DNA 2.0**	http://web.x3dna.org	3D nucleic acid structure
**web-rMKL**	https://web-rMKL.org/	Dimensionality reduction and clustering
**WebGestalt 2019**	http://www.webgestalt.org	Gene set enrichment analysis
**Yosshi**	https://biokinet.belozersky.msu.ru/yosshi	Protein disulfide engineering
**Yvis**	http://bioinfo.icb.ufmg.br/yvis/	Antibody sequence alignment visualization

**Topics**. Papers in this year’s issue fall into several broad categories: (i) DNA and RNA, (ii) genomes and genetic variants, (iii) gene set functional enrichment analysis, (iv) phylogeny analysis and visualization, (v) proteins, protein structure, binding and docking, and protein function, (vi) text mining, and (vii) miscellaneous topics.

In the DNA and RNA category, papers include **Web 3DNA 2.0** for analysis and visualization of DNA structure (cover image); **tRNAviz** for analysis of tRNA sequences; **sRNAbench and sRNAtoolbox 2019** for analysis of small RNAs; **RNA workbench 2.0**, an extensive collection of RNA analysis tools; **RNAmod** for the annotation of mRNA modifications, and **HNADOCK** for modeling RNA/DNA 3D complex structures.

Papers in the genomes category include **antiSMASH 5.0** for detection of biosynthetic gene clusters for secondary metabolites; **CPGAVAS2** and **OGDRAW** for annotation and visualization of organellar genomes (plastids and mitochondria), and the **WashU Epigenome Browser** for exploration of epigenomics data.

In the gene set functional enrichment category, resources include updates to the well-known **g:Profiler** and **WebGestalt 2019**, the new **modEnrichr**, which is specific to model organisms, and **ChEA3** for transcription factor enrichment analysis.

The phylogeny category is particularly rich this year, including **iTOL v4, Phylogeny.IO** and **Evolview v3** for annotation and visualization of phylogenetic trees; **NGPhylogeny.fr** for phylogenetic analysis, and **AutoMLST** for multi-locus sequence analysis in bacteria.

Among the papers for protein analysis is the 20th anniversary publication of **PSIPRED**, a collection of tools for structure annotation; **IntFOLD** for protein structure prediction; **LOMETS2** for threading based structure prediction; **DaReUS-Loop** for loop modeling, and **Caver Web** aimed at the identification of tunnels and channels. Papers related to improving protein structure models include **GalaxyRefine2** for protein structure refinement, and **VoroMQA** for structure model quality assessment. Among the papers focusing on protein–protein interactions are **PIZSA**, which assesses the likelihood of interactions based on interface residue contacts, and **mCSM-PPI2** that predicts the effects of mutations on protein–protein interactions. Other papers in the category include **Yosshi** for protein engineering using disulfide bonds; an update of **SwissTargetPrediction** for potential protein targets of small molecules; **PRECOG** for prediction of the protein coupling probabilities of G-protein coupled receptors, and **Aggrescan3D 2.0** for analysis of protein solubility.

In the text mining category, publications include two from researchers at the U.S. National Center for Biotechnology Information (NCBI), **PubTator Central** and **LitSense**, which seek to simplify the task of sifting through the ever expanding biomedical literature, and **GeneShot**that retrieves ranked lists of genes most relevant to a set of search terms.

Highlights among the remaining papers include **NetworkAnalyst 3.0** for network-based gene expression analysis; **EpiAlignment** that aligns genomic regions using both sequence and epigenomic information; **SPADE** for prediction of allergen IgE epitopes; **Pergola-web** that converts longitudinal behavioral data (such as circadian data) into genomic data formats for simplified analysis and visualization; **IEDB-AR** that provides new computational tools for the Immune Epitope Database, and **CHOPCHOP v3**, a CRISPR/Cas genome editing toolbox.

**Web Services**. The Web Server Issue additionally has a special section for Web Services for data and analysis, which are accessed programmatically rather than by manual interaction with a website. One paper this year from the European Molecular Biology Laboratory and the European Bioinformatics Institute, **EMBL-EBI APIs**, reports on web services for bioinformatics sequence analysis and text searching.

**Retirement of the Editor**. It has been 13 years since Rich Roberts, the former Senior Executive Editor of *Nucleic Acids Research*, asked me in 2006 to take over as Executive Editor of the Web Server Issue. With little preparation and a naive sense of what it would mean, I eagerly accepted. In the intervening years, I have learned a great deal about the role of editor for a high impact journal, honed my diplomacy in interactions with authors and reviewers, stretched myself in order to handle the high volume of proposals and manuscripts that arrive all at once (nearly 300 proposals and 120 manuscripts annually), and developed various labor saving resources and techniques to improve my efficiency. It has been an enjoyable, always demanding, but often illuminating ride.

Over the years, I have pushed to improve the quality of the Web Server publications. Websites that make predictions must now have published validation on datasets not used in training (something that, initially, many authors were surprisingly unwilling to accept, but which clearly separates higher from lower quality work). Additionally, those publications dealing with topics that are the subject of international assessment efforts must now report their results in those assessments (e.g. CASP experiments for assessment of protein folding prediction and CAFA experiments for assessment of protein function prediction). All websites must have informative help pages and provide sample data for users to try out. And use of modern methods for visualization of data is encouraged.

One aspect of the Web Server Issue that initially attracted me, and which I continue to fiercely defend and take pride in, is the insistence on open access to computational resources. Every author of a Web Server Issue proposal knows that the first requirement is a statement that says, “This website is free and open to all users and there is no login requirement.” This is in line with the Open Access publication model, adopted, in a pioneering role, by *Nucleic Acids Research* just 2 years prior to the start of my tenure.

As failures in the realm of reproducibility in science have become more common and more commonly known, and as computation has become a critical aspect of molecular biology research, I and other editors at *Nucleic Acids Research* have also worked to ensure that software reported in the journal is either freely available or preferably available as source code in an open source repository.

Finally, as I prepare to move on to other pursuits, I would like to state my firm belief that **standards matter**. Peer review matters. Reproducibility matters. Data accessibility, transparency in conflict of interest, careful editorial oversight, all of these matter. Without vigilant attention to each of these, the scientific literature will become more tainted or more self-serving, and public support for the sciences will surely erode.

**Acknowledgements**. As always, I would like to thank all those who have helped me put together the Web Server Issue. First and foremost, I would like to thank my wife, Fay Oppenheim, who has provided incomparable editorial assistance all these years. She maintains the data for all the proposals and manuscripts and handles almost all of the email interactions with reviewers. She knows an astoundingly large number of reviewers by name and reputation and is very good at predicting who will get ‘that final review’ in on time. It would have been an impossible job without her hard work and dedication.

**Figure 1.**
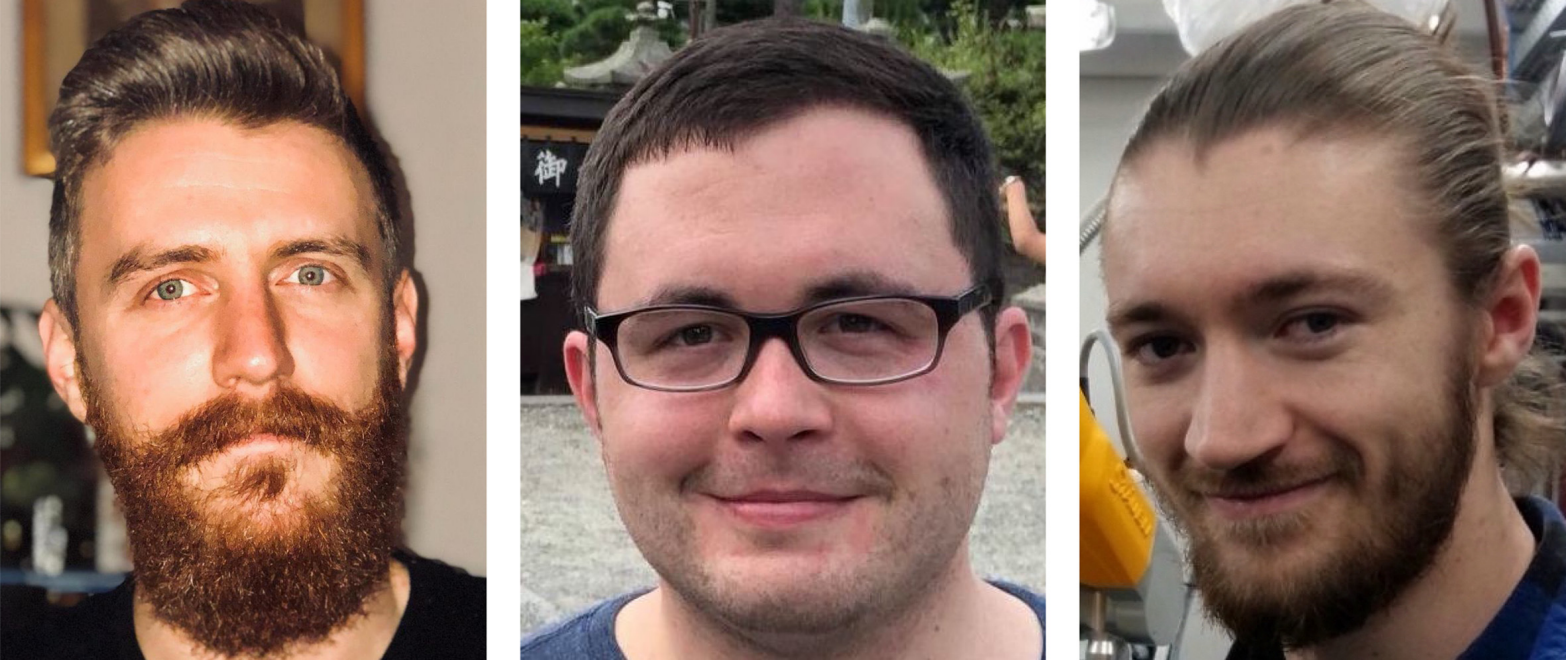
From left: Sean Corbett and David Jenkins, recent PhD graduates from the Boston University Bioinformatics Graduate Program, and Adam Simpkin, a PhD student at the Institute of Integrative Biology, University of Liverpool, UK/Synchrotron SOLEIL, France. They provided outstanding assistance in testing the 2019 Web Server proposal websites.

Next, I would like to thank the students and junior researchers who have helped me this year and in years past by testing the proposed websites to determine if they work and have required features. Testing involves many more websites than those given an okay to submit a manuscript, takes many hours, and is a task I used to do by myself. They are: Allyson Bird, Sean Corbett, Jasmin Coulombe-Huntington, Tyler Faits, Han Hu, David Jenkins, Artem Mamonov, Judith Muller, Chetanya Pandya, Joe Perez-Rogers and Adam Simpkin. Figure 1 shows the students who helped this year.

I would especially like to thank my editor colleagues who have taught me much about being a good editor. The work of an editor is done mostly in isolation, and so, it has meant a lot to me to be able to participate in our annual editors meetings, where they share their wisdom and I can feel the camaraderie of those who likewise take the job seriously.

Thanks to Dominik Seelow of the Charité - Universitätsmedizin Berlin, who will take over as Executive Editor for the Web Server Issue. He has split the job with me this year. I ask authors and reviewers to give him as much of their help and encouragement as I have received over the years. Thanks also to Martine Bernardes-Silva, Editorial Manager, NAR, and Joanna Ventikos and the staff at Oxford University Press.

Finally, I would like to thank all the authors and reviewers whose contributions and dedication have made the Web Server Issue the success that it is.

Those submitting to the 2020 Web Server Issue should be sure to check this link (https://academic.oup.com/nar/pages/submission_webserver) for any changes in deadlines and the submission email address.

Gary Benson

Executive Editor, Web Server Issue, 2007–2019

Nucleic Acids Research

New York

June 2019

